# Associations **B**etween Patient Characteristics and Cartilage T1ρ Relaxation Times Vary Over Time Following Patellar Dislocation

**DOI:** 10.1002/jor.70233

**Published:** 2026-06-09

**Authors:** John J. Elias, Lutul D. Farrow, Richard Lartey, Ahmet Hakan Ok, Mei Li, Mingrui Yang, Carl S. Winalski, Xiaojuan Li

**Affiliations:** ^1^ Cleveland Clinic Akron General Akron Ohio USA; ^2^ Cleveland Clinic Cleveland Ohio USA

**Keywords:** cartilage, contralateral, patellar dislocation, quantitative MRI, time from injury

## Abstract

The characteristics of patients that promote progressive cartilage degradation following patellar dislocations are not well understood. The study hypothesis is that associations relating cartilage degradation to low BMI, high activity and youth in the early months following patellar dislocation are not exhibited for contralateral knees or a longer time from injury. Quantitative MRI‐based evaluation of injured and contralateral knees occurred within 5 months of a unilateral patellar dislocation (baseline evaluation, 36 subjects) and beyond 5 months (follow‐up evaluation, 20 subjects). Linear regressions correlated T1ρ relaxation times for regions of patellofemoral and tibiofemoral cartilage against body mass index, age, and activity level. Long relaxation times indicate cartilage degradation. Baseline T1ρ relaxation times were significantly inversely correlated with body mass index throughout patellofemoral cartilage for injured knees (*p* < 0.001 to 0.03). Similar relationships were identified for all regions of patellar cartilage for contralateral knees (*p* = 0.011 to 0.015). Injured and contralateral T1ρ relaxation times were also correlated with activity level (positive correlation) and age (inverse correlation) for various regions of cartilage (*p* < 0.001 to 0.047). The only significant correlations for the follow‐up groups related increasing body mass index to long cartilage T1ρ relaxation times for the contralateral medial trochlear groove, femoral condyles, and lateral tibial plateau (*p* = 0.008 to 0.026). The attributes of a dislocation that determine characteristics influencing cartilage are common to both knees but dissipate over time. A systemic, transient inflammatory response to injury could explain the short‐term similarities between injured and contralateral knees following dislocation.

## Graphical abstract

MRI‐based cartilage T1ρ relaxation times were quantified for knees within 5 months of a patellar dislocation (baseline) and beyond 5 months (follow‐up), plus contralateral knees. Long T1ρ relaxation times, indicating cartilage degradation, were correlated with low BMI at the baseline evaluation for injured and contralateral knees. No significant correlations were identified for injured knees at the follow‐up evaluation. The attributes of a dislocation that determine characteristics influencing cartilage are common to both knees but dissipate over time.

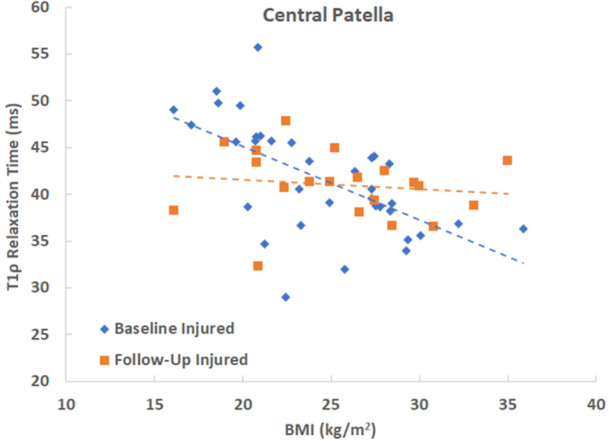

## 1 Introduction

A lateral patellar dislocation is a traumatic injury associated with early patellofemoral cartilage degradation and an elevated risk of patellofemoral osteoarthritis (OA). Adolescents experience patellar dislocations at more than twice the rate of older age groups [1]. Cartilage lesions on the medial or central patella are prevalent following patellar dislocation [2, 3, 4]. Long T1ρ and T2 relaxation times from quantitative MRI have also identified cartilage matrix degeneration shortly after a patellar dislocation, particularly for adolescents [5, 6, 7]. Approximately one‐half of knees develop symptomatic patellofemoral OA by 25 years following a patellar dislocation [8].

The characteristics of patients that contribute to progressive cartilage degradation following patellar dislocations are not well understood. High body mass index (BMI) is associated with progressive cartilage degradation to OA following traumatic knee injuries other than patellar dislocation [9, 10, 11]. Quantitative MRI has also associated high BMI with long patellofemoral and tibiofemoral cartilage T1ρ and T2 relaxation times for healthy knees and knees being treated for pain [5, 12, 13, 14, 15, 16, 17]. In the first few months following patellar dislocation, however, long patellofemoral cartilage T1ρ relaxation times have been correlated with low BMI [18]. A proposed mechanism linking low BMI to post‐traumatic cartilage degradation is a low volume of tissue surrounding the knee impairing stability and increasing traumatic impact [18, 19]. Direct trauma to cartilage and the inflammatory response to injury can both contribute to cartilage degradation [20]. Youth and high levels of activity are additional characteristics associated with low BMI related to long patellofemoral cartilage T1ρ relaxation times in the early months following patellar dislocation [18].

A better understanding of the relationship between patient characteristics and cartilage degradation is needed to determine the mechanisms that cause cartilage degradation and identify patients at highest risk of progressive degradation to OA. The current study builds on prior studies that identified parameters correlated with quantitative MRI‐based relaxation times in the early months following patellar dislocations [5, 18] by adding evaluation of contralateral knees (no direct trauma) and extending the analysis further from time of injury (reduced inflammation). Evaluation of tibiofemoral cartilage is also added. The hypothesis of the study is that associations relating cartilage degradation to low BMI, high activity and youth in the early months following patellar dislocation are not exhibited for contralateral knees or a longer time from injury.

## 2 Methods

### 2.1 Subjects/Study Groups

The study is a sub‐group analysis of subjects enrolled for a primary comparison between injured knees and healthy controls, with characteristics correlated with T1ρ relaxation times added as a supplemental analysis. Subjects were recruited based on a unilateral first‐time or recurrent dislocation of the patella from the trochlear groove within the prior 5 months. Five months is the approximate length of time cartilage recovers from the traumatic impact of a patellar dislocation [5]. A prior analysis with an overlapping population showed trends relating patient characteristics to cartilage T1ρ relaxation times were similar for first‐time and recurrent dislocations [18]. A bone bruise on the medial patella or lateral femoral condyle on MRI confirmed all dislocations. Enrollment occurred from January, 2019 to January, 2023. The study was approved by the Institutional Review Board. All subjects (plus a guardian for minors) signed a consent or assent form prior to participating.

Subjects were excluded from the study population for prior traumatic injury to either knee, other than prior patellar dislocation for the injured knee. Subjects were also excluded for prior surgery of either knee or surgery scheduled before the injured knee could be evaluated, injuries to soft tissues other than the medial retinacular structures, autoinflammatory disease, and fractures of the femur, patella or tibia other than small loose bodies. Prior MRI scans showing existing OA or moderate to large chondral or osteochondral defects that would hinder characterization of patellofemoral cartilage T1ρ relaxation times were also cause for exclusion. The analysis did not include any subjects considered severely obese (BMI ≥ 40 kg/m^2^) or with a knee otherwise considered too large to fit comfortably in the knee coil [18, 21]. Prior to October 1, 2021, the age range was 13 years and older. After that date, enrollment prioritized adolescents (13–19 years) based on data showing the most consistent differences in T1ρ relaxation times between injured knees and healthy controls occurred for adolescents [5, 6].

Injured knees and uninjured contralateral knees were evaluated at multiple time points (cohort study, level 3). Injured knees and uninjured contralateral knees within 5 months of dislocation were treated as two separate groups (baseline injured, baseline contralateral). Subjects were asked to return for a follow‐up evaluation of both knees starting at approximately 5 months from the baseline evaluation out to approximately 1 year (follow‐up injured, follow‐up contralateral). The follow up groups only included knees that did not experience an additional dislocation or undergo surgery since the baseline evaluation. To benefit the sample size, subjects initially evaluated beyond the upper baseline limit of 5 months from dislocation were included in the follow‐up group. Data collected from the subjects included date of birth, sex, BMI and date of the most recent dislocation.

### 2.2 Activity Level

Baseline and follow‐up activity levels were characterized using the Hospital for Special Surgery Pediatric Functional Activity Brief Scale (HSS Pedi‐FABS) [22]. HSS Pedi‐FABS data was collected and managed using REDCap electronic data capture tools hosted at Cleveland Clinic [23]. Frequency of activities such as running and cutting and level of athletic competition were converted to a score from 0 to 30, with 30 representing the most active.

### 2.3 Magnetic Resonance Imaging

Baseline and follow‐up MRI scans were performed on a 3 T scanner (Prisma, Siemens, Munich, Germany) using a 1Tx/15Rx knee coil (Quality Electrodynamics, Mayfield Village, OH). Cartilage surfaces were segmented and reconstructed from 3D fat saturated Dual Echo Steady State (DESS) scans (repetition time = 17.55 ms; echo time = 6.02 ms; frequency × phase = 384 × 307; slice thickness = 0.7 mm; pixel spacing = 0.365 × 0.365 mm; skip = 0 mm; flip angle = 25°). T1ρ relaxation times throughout the cartilage were quantified from a 3D fat saturated T1ρ Magnetization‐prepared Angle‐modulated Partitioned‐k‐space Spoiled gradient‐echo Snapshots (MAPSS) imaging sequence using previously described imaging parameters (spin lock times = 0, 10, 30, 70 ms; spin lock frequency = 500 Hz; repetition time = 6.37 ms; echo time = 2.9 ms; frequency × phase = 320 × 160; slice thickness = 4 mm; pixel spacing = 0.4375 × 0.4375 mm; skip = 0 mm; variable flip angle) [5, 6, 18]. To minimize the influence of cartilage compression on relaxation times [24, 25, 26], subjects were seated to complete paperwork for approximately 20 min prior to the MRI session. Subjects spent an additional 25–30 min supine prior to initiation of the MAPSS sequence.

### 2.4 Cartilage Mapping

**Figure 1:**
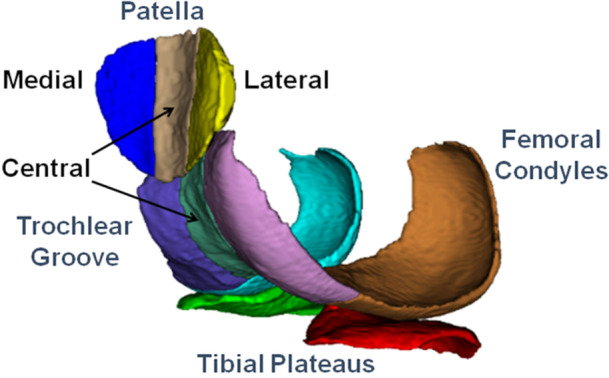
Three‐dimensional reconstruction of the patellofemoral and tibiofemoral regions of cartilage from one knee model.

T1ρ relaxation times were mapped to regions of patellofemoral and tibiofemoral cartilage. Cartilage was evaluated within regions representing the medial, central and lateral patella and trochlear groove (Figure 1). Cartilage was additionally evaluated within regions representing the medial and lateral femoral condyles and tibial plateaus. Borders of the cartilage at the articular surface and underlying bone were identified on the DESS scans using a previously trained deep learning model that combines conditional generative adversarial networks and convolutional neural networks [27]. Manual correction of cartilage borders was applied as needed. The trochlear groove was separated from the femoral condyles based on Blumensaat's line along the roof of the intercondylar notch projected medially and laterally through the femur. An automated closed‐contour mapping algorithm identified the most medial and lateral points on the patella and most prominent point on the patellar ridge from axial slices through the patella. The patella was divided into medial, central and lateral regions at points one‐third of the distance from the central ridge to the medial and lateral edges of the patella [5, 6]. The trochlear groove was similarly divided into medial, central and lateral regions based on points at the medial and lateral edges of cartilage and the deepest point of the trochlear groove. Manual correction of automatically selected points was applied as needed.

**Figure 2:**
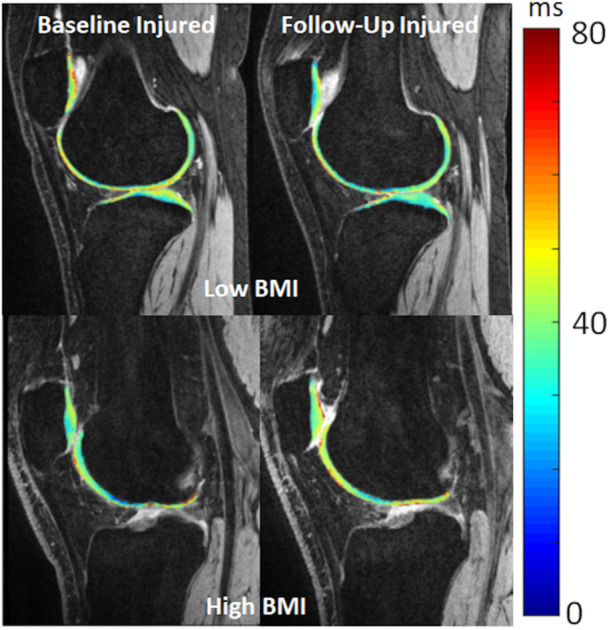
T1ρ relaxation times mapped to cartilage for injured knees from low BMI (16 kg/m^2^) and high BMI (32 kg/m^2^) subjects at the baseline and follow‐up evaluations.

The first echo of the T1ρ images was rigidly registered with images from the DESS scan, using piecewise rigid registration, which aligned the images while minimizing variations related to movement of the subject [5, 6]. To enhance the rigid registration of the remaining T1ρ echoes to the first echo, bone masks were transformed from a knee template to the first echo after non‐linear registration. These transformed masks helped to correct for small movements within the T1ρ sequence. A two parameter, monoexponential Levenberg‐Marquardt algorithm related image signal to exponential decay based on time of spin lock to T1ρ relaxation time. T1ρ relaxation times were mapped to each voxel within the cartilage borders (Figure 2). Extreme relaxation times (> 200 ms) were discarded to avoid partial volume averaging effects. T1ρ relaxation times were averaged for each region of cartilage. Scan–rescan root mean square coefficients of variation for T1ρ relaxation times have been reported as less than 5% for regions of cartilage including the whole patella and trochlear groove [28, 29]. Scan‐rescan root mean square coefficients of variation for T1ρ relaxation times remained below 5% when the sub‐divisions were applied to evaluate medial, central, and lateral regions of cartilage on the patella and within the trochlear groove.

### 2.5 Statistical Analysis

Characteristics of the subjects were compared between groups to examine for differences that could influence cartilage relaxation times. Age, BMI, and activity level were compared between the groups. Time from most recent dislocation was pre‐determined to differ between the baseline and follow‐up groups. The level of significance was *p* < 0.05 for each analysis. Normality of the dependent variables was evaluated with a Shapiro‐Wilk test and homogeneity of variances was evaluated with a Levene's test (SPSS version 24, IBM, Armonk, NY) to check the assumptions for parametric analyses. ANOVA was used for parametric analyses (*t*‐test for two group comparisons), and Kruskall‐Wallis tests (Mann‐Whitney U test for two group comparisons) were used for nonparametric analyses. Distribution of sex and single versus multiple dislocations were compared between the groups with a Chi square test after checking the expected frequency for all cells.

For each region of patellofemoral and tibiofemoral cartilage, T1ρ relaxation times were correlated against BMI, age, time from latest dislocation and activity level. The regressions considered four groups: baseline injured, baseline contralateral, follow‐up injured and follow‐up contralateral. The groups were treated separately based on the expectation that relationships between T1ρ relaxation times and characteristics of the subjects would vary between groups, rather than expectation of consistent relationships with T1ρ relaxation times across groups. A stratified approach was used to identify differences between groups that might not be fully captured with interaction terms from a higher order model. Shapiro‐Wilk tests for normality of unstandardized residuals and White tests for heteroscedasticity of variances of the residuals were used to check the assumptions for linear regressions. Multivariable linear regressions were performed to evaluate the relative contribution of each independent variable when multiple variables were significantly correlated with T1ρ relaxation times. Variance influence factors were quantified to rule out multicollinearity. Non‐normal distributions of the residuals were addressed with Spearman rank order correlations, and heteroscedasticity was addressed with weighted least squares regressions. Coefficients of determination (r^2^) were quantified, and standardized beta (β) coefficients provided measures of the strength and direction for linear regressions. A power analysis for the baseline evaluations was developed from prior data relating characteristics of subjects to T1ρ relaxation times following patellar dislocations [18], with the goal of reaching a coefficient of determination exceeding 0.25 for a sample size of 35 to produce a power of 0.9. The goal for follow up was to re‐evaluate at least 50% of the baseline subjects, with the goal of reaching a coefficient of determination exceeding 0.36 to produce a power of 0.85. T1ρ relaxation times were compared between males and females with t‐tests or Mann‐Whitney U‐tests, as appropriate. When significant differences were identified based on sex, linear regressions for T1ρ relaxation times were repeated including an interaction term between sex and other significant subject characteristics.

## 3 Results

**Figure 3:**
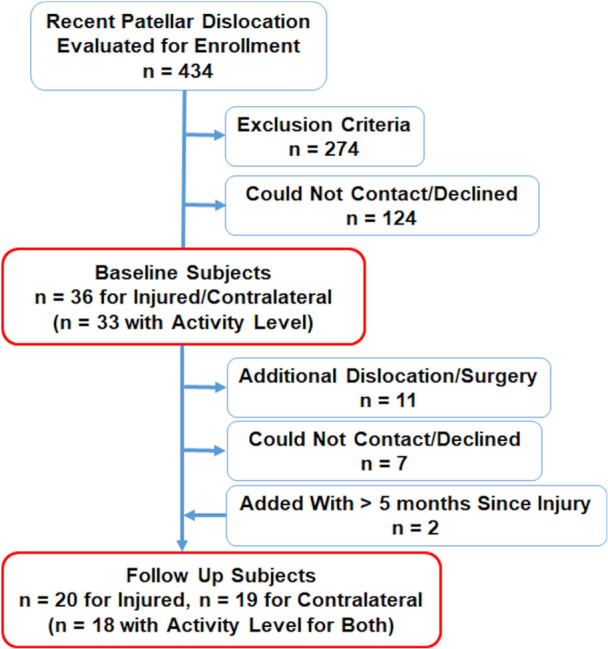
Flowchart showing enrollment of patellar dislocation patients.

The study population consisted of 36 subjects for the baseline injured and contralateral groups (Figure 3). Twenty subjects participated in the follow‐up evaluation, although only the injured knee was evaluated for one subject. The patient population was drawn from 434 patients evaluated within 5 months of a patellar dislocation, with 274 excluded based on one or more exclusion criteria. The baseline group included 23 subjects evaluated after a first dislocation and 13 after multiple dislocations. The follow‐up group included 16 subjects evaluated after a first dislocation and 4 evaluated after multiple dislocations. Of the 36 subjects evaluated at the baseline evaluation, 11 were excluded from the follow‐up evaluation for additional dislocations or surgery. Two subjects were added to the follow‐up group without data for the baseline group. Of the 36 and 20 subjects in the baseline and follow‐up groups, activity level surveys were completed by 33 and 18 subjects, respectively. The subject missing contralateral evaluation for the follow‐up group was one of the two missing activity level.

Subject characteristics were similar between the baseline and follow‐up groups. No significant differences in age, BMI, activity level, distribution of sex or distribution of single versus multiple dislocations were identified between the baseline and follow‐up groups (*p* > 0.3, Table 1). The average time from dislocation to evaluation was approximately 2 months (57 ± 36 days) for the baseline injured group and 9 months (264 ± 66) for the follow‐up injured group.

**Table 1:** Demographic characteristics for the groups. Data for the baseline injured and contralateral groups are identical. Continuous data are presented as mean ± standard deviation.

	Baseline injured/contralateral	Follow‐up injured	Follow‐up contralateral	*p*‐value
Sex (male/female)	14/22	10/10	9/10	0.68
Dislocations (single/multiple)	23/13	16/4	15/4	0.32
Age (years)	19.5 ± 6.5	20.5 ± 7.1	20.7 ± 7.2	0.38^b^
Body mass index (kg/m^2^)	24.5 ± 4.5	25.6 ± 4.9	25.5 ± 5.0	0.64
Activity level (HSS Pedi‐FABS)	11.3 ± 12.0	13.7 ± 12.8	13.7 ± 12.8	0.65^a^
Time since recent dislocation (days)	57 ± 36	264 ± 66	267 ± 66	

a Mann Whitney U‐test.

^b^ Kruskall‐Wallis test.

**Figure 4:**
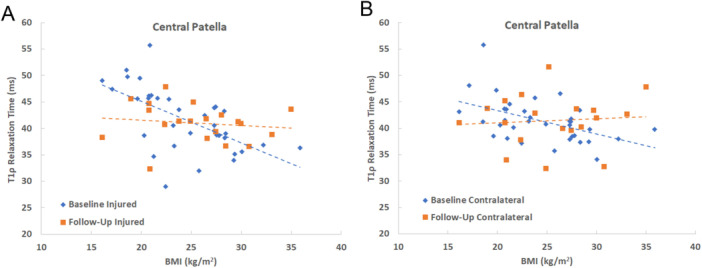
Baseline and follow‐up cartilage T1ρ relaxation times at the central patella related to body mass index (BMI) for the injured knees (A) and contralateral knees (B).

For the baseline injured and contralateral groups, long cartilage T1ρ relaxation times were related to a low BMI, young age, and high activity (Table 2). T1ρ relaxation times were significantly inversely correlated with BMI for all regions on the patella for injured and contralateral knees (*p* < 0.001 to 0.03, Figure 4). Significant correlations between T1ρ relaxation times and BMI were also identified for all regions of the trochlear groove for the injured group (*p* < 0.001 to 0.013). T1ρ relaxation times were positively correlated with activity level at the lateral femoral condyle (*p* = 0.009) and lateral tibia (*p* = 0.044) for the injured group and at the central patella, medial and central trochlear groove, and medial and lateral femoral condyle for the contralateral group (*p* = 0.006 to 0.041). T1ρ relaxation times were inversely correlated with age at the central trochlear groove for the injured group (*p* = 0.047) and at the lateral trochlear groove for the contralateral group (*p* < 0.001). T1ρ relaxation times were also inversely correlated with time since latest dislocation at the medial patella (*p* = 0.009) but positively correlated with time since latest dislocation at the medial femoral condyle (*p* = 0.027) for the injured group. Average T1ρ relaxation times for each region of cartilage for each group are provided in Table S1.

**Table 2:** Significant correlations relating T1ρ relaxation times (msec) to subject characteristics. No significant correlations were identified for the follow‐up injured group.

	Parameter	*r* ^2^	*p*‐value	Standardized β
Baseline injured				
Medial patella	BMI (kg/m^2^)	0.30^a^^,^ b	0.030	−0.33
	Time Since Dislocation (days)		0.009	−0.41
Central patella	BMI (kg/m^2^)	0.45^c^	< 0.001	
Lateral patella	BMI (kg/m^2^)	0.32^a^	< 0.001	−0.57
Medial trochlear groove	BMI (kg/m^2^)	0.38^a^	< 0.001	−0.62
Central trochlear groove	BMI (kg/m^2^)	0.35^a^^,^ b	0.013	−0.40
	Age (years)		0.047	−0.32
Lateral trochlear groove	BMI (kg/m^2^)	0.25	0.002	−0.50
Medial femoral condyle	Time Since Dislocation (days)	0.14^c^	0.027	
Lateral femoral condyle	Activity Level	0.20^c^	0.009	
Lateral tibia	Activity Level	0.12	0.044	0.35
Baseline contralateral				
Medial patella	BMI (kg/m^2^)	0.16^a^	0.015	−0.40
Central patella	BMI (kg/m^2^)	0.36^a^^,^ b	0.011	−0.41
	Activity Level		0.026	0.35
Lateral patella	BMI (kg/m^2^)	0.16^a^	0.015	−0.40
Medial trochlear groove	Activity Level	0.19^a^	0.012	0.43
Central trochlear groove	Activity Level	0.22^a^	0.006	0.47
Lateral trochlear groove	Age (years)	0.30^a^	< 0.001	−0.55
Medial femoral condyle	Activity Level	0.13^a^	0.041	0.36
Lateral femoral condyle	Activity Level	0.18^c^	0.013	
Follow‐up contralateral				
Medial trochlear groove	BMI (kg/m^2^)	0.26^a^	0.026	0.51
Medial femoral condyle	BMI (kg/m^2^)	0.35	0.008	0.59
Lateral femoral condyle	BMI (kg/m^2^)	0.34^a^	0.009	0.58
Lateral tibia	BMI (kg/m^2^)	0.30^a^	0.016	0.54

a Weighted least squares regression.

^b^ r^2^ for multivariable regression.

^c^ Spearman correlation.

**Figure 5:**
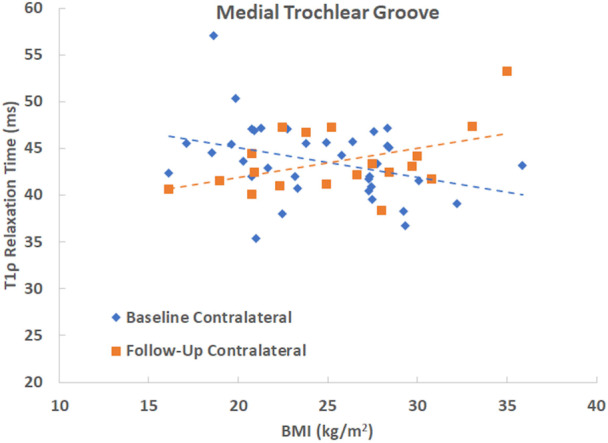
Baseline and follow‐up cartilage T1ρ relaxation times at the medial trochlear groove related to body mass index (BMI) for the contralateral knees.

The only significant correlations for the follow‐up groups related increasing BMI to long cartilage T1ρ relaxation times for the contralateral group. No significant correlations were identified for the follow‐up injured group (*p* > 0.1). For the follow‐up contralateral group, T1ρ relaxation times were significantly correlated with high BMI for the medial trochlear groove, medial and lateral femoral condyles, and lateral tibial plateau (*p* = 0.008 to 0.026, Figure 5).

T1ρ relaxation times differed based on sex for only a few comparisons. T1ρ relaxation times were significantly longer for males than females at the medial femoral condyle for the follow‐up injured group (45.8 ± 4.0 vs. 42.3 ± 2.5, *p* = 0.031) and the baseline contralateral group (47.4 ± 5.3 vs. 43.7 ± 2.3, *p* = 0.024). T1ρ relaxation times were significantly longer for females than males at the central patella for the baseline contralateral group (41.9 ± 2.9 vs. 40.4 ± 5.4, *p* = 0.035 from Mann‐Whitney U‐test). For these conditions with significant differences, no significant interactions between sex and other patient characteristics were identified (*p* > 0.1). No significant relationships based on sex were identified for the baseline injured or follow‐up contralateral groups (*p* > 0.07).

## 4 Discussion

The results show similar relationships between patient characteristics and cartilage T1ρ relaxation times for injured and contralateral knees in the early months following patellar dislocation. Low BMI, young age, and high activity levels were expected to be related to long cartilage T1ρ relaxation times for the baseline injured group based on similar results for an overlapping population that evaluated single and multiple dislocations separately [18]. The relationships between T1ρ relaxation times and low BMI, young age, and high activity levels for the baseline contralateral group contradicts the hypothesis. The results for the baseline contralateral group indicate factors besides direct traumatic impact influence how cartilage of both knees respond to traumatic injury. For the baseline injured group, the parameter most strongly correlated with long patellofemoral relaxation times was low BMI. For the baseline contralateral group, the parameter most strongly correlated with long relaxation times was low BMI for cartilage on the patella but high activity for trochlear groove cartilage. High activity was also the parameter most strongly correlated with long tibiofemoral cartilage relaxation times for the injured and contralateral groups. A prior study with an overlapping population showed trends for elevated tibiofemoral cartilage T1ρ relaxation times shortly following a first‐time patellar dislocation [5]. Similar factors related to T1ρ relaxation times for patellofemoral and tibiofemoral cartilage further indicate that conditions besides direct traumatic impact influence how cartilage responds to traumatic injury.

The relationships between patient characteristics and cartilage T1ρ relaxation times were not maintained from baseline to follow‐up. For the follow‐up injured group, no patient characteristics were significantly correlated with T1ρ relaxation times. For the follow‐up contralateral group, T1ρ relaxation times increased with BMI for the medial trochlear groove and three tibiofemoral regions, generally reversing baseline trends. T1ρ relaxation times increasing with BMI aligns with the influence of BMI on patellofemoral and tibiofemoral cartilage relaxation times for healthy knees [12, 13, 18, 30]. For contralateral knees, the attributes of patellar dislocation inducing relationships between patient characteristics and cartilage relaxation times that are dissimilar to healthy knees may have dissipated following the baseline evaluation. For injured and contralateral knees, the baseline and follow‐up trendlines crossed at a BMI of approximately 25 kg/m^2^ (Figures 4, 5), which is the boundary separating normal and overweight categories for BMI. The crossing point gives an indication that relatively long patellofemoral cartilage T1ρ relaxation times in the first few months following a dislocation are primarily experienced by patients with a low to normal BMI (Figure 2).

Prior studies have related BMI, activity level and age to cartilage degradation following knee injuries. For traumatic knee injuries other than patellar dislocation, progressive cartilage degradation to OA has primarily been related to a BMI considered overweight [9, 10, 11], although one prior study showed a quadratic relationship between BMI and knee cartilage characteristics within 3 years of ACL reconstruction [19]. Activity levels considered higher and lower than moderate have both been related to elevated knee cartilage T1ρ and T2 relaxation times for healthy knees and knees at risk of OA due to pain or an injury [25, 31, 32, 33]. Existing literature relating age to cartilage T1ρ and T2 relaxation times has been inconsistent. For age ranges overlapping with the current study, increasing and decreasing relaxation times have been reported as age increases for healthy knees [5, 24, 34, 35] and knees being treated for injuries [36, 37]. For longer term cartilage degradation, young age at the time of patellar dislocation has been specifically related to patellofemoral OA [8]. Based on the existing literature and current data, long term progression to OA could be influenced by factors influencing cartilage in the early months following patellar dislocation or factors associated with cartilage degradation for healthy knees. Prior studies have not addressed the possibility of parameters influencing cartilage properties changing over time following patellar dislocation.

T1ρ relaxation times decreased over the first 5 months from injury at the medial patella, demonstrated by a significant correlation with time from most recent dislocation for only the baseline injured group. Prior studies with an overlapping population similarly showed T1ρ relaxation times for cartilage on the medial patella decrease over time following a first dislocation [5, 18]. The medial patella is the site of traumatic impact against the lateral femoral condyle during a dislocation episode. Based on the significant correlation with time from injury for the baseline injured group but not the baseline contralateral group, cartilage at the medial patella likely undergoes a recovery phase following direct traumatic impact. Based on lack of a relationship between T1ρ relaxation times and time from injury for cartilage on the medial patella for the follow‐up injured group, this recovery period likely does not extend much beyond 5 months. The only region with cartilage T1ρ relaxation times increasing with time from injury was the medial femoral condyle for the baseline injured group, but this relationship did not hold for the follow‐up injured group.

T1ρ relaxation times were longer for males than females at the medial femoral condyle for the follow‐up injured group and baseline contralateral group but longer for females than males at the central patella for the baseline contralateral group. The variations due to sex did not produce significant interactions with other characteristics, and do not alter the primary characteristics related to T1ρ relaxation times for each group. While the current study shows no significant influence of sex for the baseline injured group, a prior study with an overlapping population showed significantly longer T1ρ relaxation times for females than males for cartilage on the patella for knees being treated for a first dislocation and significantly longer T1ρ relaxation times for males than females for trochlear groove cartilage for knees being treated for multiple dislocations [18]. Relationships between sex and cartilage relaxation times likely vary with number of dislocations, time from injury and region of cartilage.

The mechanism relating low BMI, young age, and high activity to long patellofemoral and tibiofemoral cartilage T1ρ relaxation times in the early months following patellar dislocation is currently unknown. Relatively low volume of tissue at the knee could contribute to functional instability or elevate the traumatic impact and inflammatory response during a patellar dislocation, both of which can contribute to early cartilage degradation [5, 6, 20]. Because the baseline correlations for contralateral knees are similar but generally weaker than those for injured knees, a systemic inflammatory response to injury may be influencing the patient characteristics related to cartilage properties. Prior studies have shown evidence of systemic inflammation following knee injuries and arthroscopic knee surgery [38, 39] with the influence weaker for the contralateral than the injured knee. Recovery from traumatic impact and altered function following dislocation could also influence the patient characteristics related to cartilage properties, but these features are less likely to produce similar effects for both knees. Further studies on the mechanisms that initiate cartilage degradation and promote progressive degradation to post‐traumatic OA are needed to develop robust cartilage preservation strategies following patellar dislocation.

The primary limitations are related to the study population and time points for evaluation. This sub‐group analysis of a larger patellar dislocation cohort includes subjects originally recruited before and after implementing a maximum age of 19 years based on larger differences between injured and healthy knees for adolescents than older subjects [5, 6]. The statistical analysis addressed heteroscedasticity in the population with weighted least squared regressions. The analysis included three parameters generally related to each other for the young population, but multicollinearity between age, BMI and activity level was not identified for any significant relationships. Consistency of time from injury for the baseline and follow‐up evaluations and rates of successful enrollment and follow‐up were limited by common constraints when evaluating injured subjects, such as time needed to identify and contact subjects, distance from home or school to the imaging center, and limited availability for the subjects. The enrollment and follow‐up periods also partially overlapped with the Covid‐19 pandemic.

Other limitations are related to the data collected. BMI does not account for proportion or distribution of fat or proportion of muscle mass. Future analyses may benefit from more accurate measures, such as waist to hip ratio or body fat percentage [40, 41]. The HSS Pedi‐FABS survey could underrepresent activity levels for non‐athletes and athletes in sports with limited running and change of direction [22]. The study focused on characteristics of the whole subject, without including knee anatomy or alignment. A prior study including an overlapping population examined measures of anatomy and alignment following a first dislocation and multiple dislocations [6]. The only significant correlations related long T1ρ relaxation times to patellar height (central and lateral trochlear groove) and lateral shift (medial patella) following a first‐time dislocation. Additional studies with a larger population are warranted to evaluate interactions between anatomy, alignment and characteristics of the whole subject. Cartilage was evaluated based only on T1ρ relaxation times related to proteoglycan concentration. Quantifying T2 relaxation times could also assess the cartilage matrix based on water and collagen content, along with organization of collagen fibrils.

## 5 Conclusion

In the early months following a patellar dislocation, low BMI, young age, and high activity are related to long patellofemoral and tibiofemoral cartilage T1ρ relaxation times for injured and contralateral knees. Similar results for injured and contralateral knees indicate the attributes of patellar dislocation that determine the characteristics influencing cartilage are common to both knees rather than isolated to the traumatic injury. Beyond the first several months following dislocation, the attributes related to cartilage degradation shift to high BMI for contralateral knees. A systemic, transient inflammatory response to injury could potentially contribute to similarities between injured and contralateral knees over the early months following dislocation and a transition in the factors influencing cartilage beyond the first few months.

## Author Contributions

John Elias participated in study design, recruitment, imaging, image processing, data analysis, statistical analysis and manuscript preparation. Lutul Farrow participated in study design, recruitment, and data analysis. Richard Lartey participated in development of MRI sequences and imaging processing protocols and contributed to image processing. Ahmet Hakan Ok participated in image processing for segmentation of cartilage and T1ρ relaxation time mapping. Mei Li participated in study oversight, image processing for segmentation of cartilage and T1ρ relaxation time mapping. Mingrui Yang participated in development of imaging processing protocols and data analysis. Carl Winalski participated in development of image processing protocols, analysis of MRI and data analysis. Xiaojuan Li participated in study design, development of MRI sequences and imaging processing protocols, data analysis and manuscript revisions. All authors have read and approved the final submitted manuscript.

## Supporting information


**Table S1:** Average (± standard deviation) T1ρ relaxation times for all regions of cartilage for all groups.

## Data Availability

The data that support the findings of this study are available from the corresponding author upon reasonable request.

## References

[1] T. L. Sanders , A. Pareek , T. E. Hewett , M. J. Stuart , D. L. Dahm , and A. J. Krych , “Incidence of First‐Time Lateral Patellar Dislocation: A 21‐Year Population‐Based Study,” Sports Health: A Multidisciplinary Approach 10 (2018): 146–151. 10.1177/1941738117725055 PMC5857724 28795924

[2] E. Nomura , M. Inoue , and M. Kurimura , “Chondral and Osteochondral Injuries Associated With Acute Patellar Dislocation,” Arthroscopy 19 (2003): 717–721. 12966379 10.1016/s0749-8063(03)00401-8

[3] E. E. Salonen , T. Magga , P. J. Sillanpää , T. Kiekara , H. Mäenpää , and V. M. Mattila , “Traumatic Patellar Dislocation and Cartilage Injury: A Follow‐up Study of Long‐Term Cartilage Deterioration,” American Journal of Sports Medicine 45 (2017): 1376–1382. 28298062 10.1177/0363546516687549

[4] B. Vollnberg , T. Koehlitz , T. Jung , et al., “Prevalence of Cartilage Lesions and Early Osteoarthritis in Patients With Patellar Dislocation,” European Radiology 22 (2012): 2347–2356. 22645041 10.1007/s00330-012-2493-3

[5] J. J. Elias , M. Li , M. Yang , et al., “Elevated Patellofemoral and Tibiofemoral T1rho Relaxation Times Following a First Time Patellar Dislocation,” Cartilage 13 (2022): 19476035221102570. 35676874 10.1177/19476035221102570 PMC9189536

[6] L. D. Farrow , J. J. Elias , M. Li , et al., “Patellar Dislocation in Adolescent Patients: Influence on Cartilage Properties Based on T1ρ Relaxation Times,” American Journal of Sports Medicine 51 (2023): 3714–3723. 37897349 10.1177/03635465231205562 PMC11087140

[7] E. Voronkova , I. Melnikov , A. Manzhurtsev , et al., “T(2) Mapping of Patellar Cartilage After a Single First‐Time Episode of Traumatic Lateral Patellar Dislocation,” Journal of Magnetic Resonance Imaging 59 (2023): 865–876. 37316971 10.1002/jmri.28857

[8] T. L. Sanders , A. Pareek , N. R. Johnson , M. J. Stuart , D. L. Dahm , and A. J. Krych , “Patellofemoral Arthritis After Lateral Patellar Dislocation: A Matched Population‐Based Analysis,” American Journal of Sports Medicine 45 (2017): 1012–1017. 28005405 10.1177/0363546516680604

[9] M. K. Group , M. H. Jones , S. R. Oak , et al., “Predictors of Radiographic Osteoarthritis 2 to 3 Years After Anterior Cruciate Ligament Reconstruction: Data From the MOON On‐Site Nested Cohort,” Orthopaedic Journal of Sports Medicine 7 (2019): 2325967119867085. 31516911 10.1177/2325967119867085 PMC6719483

[10] C. A. Jacobs , C. E. W. Conley , D. L. Johnson , D. C. Landy , and A. V. Stone , “Leveraging Insurance Claims Data to Identify Risk Factors for Posttraumatic Osteoarthritis After Multiligament Knee Reconstruction,” American Journal of Sports Medicine 51 (2023): 1491–1496. 37014296 10.1177/03635465231162105

[11] S. G. Bodkin , B. C. Werner , L. V. Slater , and J. M. Hart , “Post‐Traumatic Osteoarthritis Diagnosed Within 5 Years Following ACL Reconstruction,” Knee Surgery, Sports Traumatology, Arthroscopy 28 (2020): 790–796. 10.1007/s00167-019-05461-y 30887068

[12] K. S. Tamayo , L. N. Heckelman , C. E. Spritzer , L. E. DeFrate , and A. T. Collins , “Obesity Impacts the Mechanical Response and Biochemical Composition of Patellofemoral Cartilage: An In Vivo, MRI‐Based Investigation,” Journal of Biomechanics 134 (2022): 110991. 35176590 10.1016/j.jbiomech.2022.110991 PMC11103252

[13] A. T. Collins , M. L. Kulvaranon , H. C. Cutcliffe , et al., “Obesity Alters the In Vivo Mechanical Response and Biochemical Properties of Cartilage as Measured by MRI,” Arthritis Research & Therapy 20 (2018): 232. 30333058 10.1186/s13075-018-1727-4 PMC6235204

[14] Z. Wang , S. Ai , F. Tian , et al., “Higher Body Mass Index Is Associated With Biochemical Changes in Knee Articular Cartilage After Marathon Running: A Quantitative T2‐Relaxation MRI Study,” Orthopaedic Journal of Sports Medicine 8 (2020): 2325967120943874. 32851106 10.1177/2325967120943874 PMC7427140

[15] G. B. Joseph , C. E. McCulloch , M. C. Nevitt , et al., “A Reference Database of Cartilage 3 T MRI T2 Values in Knees Without Diagnostic Evidence of Cartilage Degeneration: Data From the Osteoarthritis Initiative,” Osteoarthritis and Cartilage 23 (2015): 897–905. 25680652 10.1016/j.joca.2015.02.006 PMC4444394

[16] J. Verschueren , S. J. Van Langeveld , J. L. Dragoo , et al., “T2 Relaxation Times of Knee Cartilage in 109 Patients With Knee Pain and Its Association With Disease Characteristics,” Acta Orthopaedica 92 (2021): 335–340. 33538221 10.1080/17453674.2021.1882131 PMC8231385

[17] M. F. Koff , K. K. Amrami , and K. R. Kaufman , “Clinical Evaluation of T2 Values of Patellar Cartilage in Patients With Osteoarthritis,” Osteoarthritis and Cartilage 15 (2007): 198–204. 16949313 10.1016/j.joca.2006.07.007

[18] J. J. Elias , L. D. Farrow , R. Lartey , et al., “Patellofemoral Cartilage Degradation Based on T1rho Relaxation Times Varies Inversely With BMI After Patellar Dislocation,” Orthopaedic Journal of Sports Medicine 13 (2025): 23259671251334634. 40401089 10.1177/23259671251334634 PMC12092981

[19] F. F. Altahawi , E. K. Reinke , I. Briskin , et al., “Meniscal Treatment as a Predictor of Worse Articular Cartilage Damage on MRI at 2 Years After ACL Reconstruction: The MOON Nested Cohort,” American Journal of Sports Medicine 50 (2022): 951–961. 35373606 10.1177/03635465221074662 PMC9176689

[20] B. J. Evers , M. H. J. Van Den Bosch , A. B. Blom , P. M. van der Kraan , S. Koëter , and R. M. Thurlings , “Post‐Traumatic Knee Osteoarthritis; the Role of Inflammation and Hemarthrosis on Disease Progression,” Frontiers in Medicine 9 (2022): 973870. 36072956 10.3389/fmed.2022.973870 PMC9441748

[21] R. Bhattacharjee , E. Hammond , N. Chotigar , et al., “The Relationships between Patellofemoral Bone Remodeling, Cartilage Composition, and Vertical Loading Rate: PET/MRI in Isolated Patellofemoral Osteoarthritis,” Osteoarthritis and Cartilage 32 (2024): 1591–1600. 39277026 10.1016/j.joca.2024.09.001 PMC11781082

[22] P. D. Fabricant , A. Robles , T. Downey‐Zayas , et al., “Development and Validation of a Pediatric Sports Activity Rating Scale: The Hospital for Special Surgery Pediatric Functional Activity Brief Scale (HSS Pedi‐FABS),” American Journal of Sports Medicine 41 (2013): 2421–2429. 23893420 10.1177/0363546513496548

[23] P. A. Harris , R. Taylor , R. Thielke , J. Payne , N. Gonzalez , and J. G. Conde , “Research Electronic Data Capture (REDCap)—A Metadata‐Driven Methodology and Workflow Process for Providing Translational Research Informatics Support,” Journal of Biomedical Informatics 42 (2009): 377–381. 18929686 10.1016/j.jbi.2008.08.010 PMC2700030

[24] E. Wellsandt , J. Emory , Y. M. Golightly , et al., “Individual and Cumulative Measures of Knee Joint Load Associate With T2 Relaxation Times of Knee Cartilage in Young, Uninjured Individuals: A Pilot Study,” Knee 32 (2021): 19–29. 34371371 10.1016/j.knee.2021.07.004

[25] J. M. Friedman , F. Su , A. L. Zhang , et al., “Patient‐Reported Activity Levels Correlate With Early Cartilage Degeneration After Anterior Cruciate Ligament Reconstruction,” American Journal of Sports Medicine 49 (2021): 442–449. 33395319 10.1177/0363546520980431

[26] M. S. White , S. A. Garcia , Y. Pang , C. M. Casey , R. M. Palmieri‐Smith , and L. K. Lepley , “Patellofemoral Cartilage Changes Are Not Associated With Quadriceps Metrics After ACLR With Patellar Tendon Autografts,” Journal of Orthopaedic Research 43 (2025): 1432–1441. 40400186 10.1002/jor.26102

[27] S. Gaj , M. Yang , K. Nakamura , and X. Li , “Automated Cartilage and Meniscus Segmentation of Knee MRI With Conditional Generative Adversarial Networks,” Magnetic Resonance in Medicine 84 (2020): 437–449. 31793071 10.1002/mrm.28111

[28] J. Kim , K. Mamoto , R. Lartey , et al., “Multi‐Vendor Multi‐Site T1ρ and T2 Quantification of Knee Cartilage,” Osteoarthritis and Cartilage 28 (2020): 1539–1550. 32739341 10.1016/j.joca.2020.07.005 PMC8094841

[29] X. Li , V. Pedoia , D. Kumar , et al., “Cartilage T1ρ and T2 Relaxation Times: Longitudinal Reproducibility and Variations Using Different Coils, MR Systems and Sites,” Osteoarthritis and Cartilage 23 (2015): 2214–2223. 26187574 10.1016/j.joca.2015.07.006 PMC4663102

[30] S. Lehtovirta , A. Kemppainen , M. Haapea , et al., “Effects of Bariatric Surgery on Knee Articular Cartilage and Osteoarthritis Symptoms‐A 12‐Month Follow‐Up Using T2 Relaxation Time and WOMAC Osteoarthritis Index,” Journal of Magnetic Resonance Imaging 60 (2024): 2433–2444. 38558426 10.1002/jmri.29369

[31] D. Jandacka , V. Casula , J. Hamill , et al., “Regular Running Is Related to the Knee Joint Cartilage Structure in Healthy Adults,” Medicine & Science in Sports & Exercise 56 (2024): 1026–1035. 38233979 10.1249/MSS.0000000000003386

[32] W. Lin , H. Alizai , G. B. Joseph , et al., “Physical Activity in Relation to Knee Cartilage T2 Progression Measured With 3 T MRI Over a Period of 4 Years: Data From the Osteoarthritis Initiative,” Osteoarthritis and Cartilage 21 (2013): 1558–1566. 23831632 10.1016/j.joca.2013.06.022 PMC3874212

[33] K. K. Hovis , C. Stehling , R. B. Souza , et al., “Physical Activity Is Associated With Magnetic Resonance Imaging‐Based Knee Cartilage T2 Measurements in Asymptomatic Subjects With and Those Without Osteoarthritis Risk Factors,” Arthritis & Rheumatism 63 (2011): 2248–2256. 21538328 10.1002/art.30419 PMC3149726

[34] J. C. Nguyen , H. Allen , F. Liu , K. M. Woo , Z. Zhou , and R. Kijowski , “Maturation‐Related Changes in T2 Relaxation Times of Cartilage and Meniscus of the Pediatric Knee Joint at 3 T,” American Journal of Roentgenology 211 (2018): 1369–1375. 30299996 10.2214/AJR.18.20026 PMC6314193

[35] K. Aschauer , M. A. Weber , R. Bülow , et al., “Association of Layer‐Specific Knee Cartilage T2‐relaxation Measurements With Age, Sex and Cartilage Morphology at 1.5‐T MRI,” European Radiology 36 (2026): 308–323. 40715824 10.1007/s00330-025-11806-8 PMC12711982

[36] K. Amano , J. L. Huebner , T. V. Stabler , et al., “Synovial Fluid Profile at the Time of Anterior Cruciate Ligament Reconstruction and Its Association With Cartilage Matrix Composition 3 Years After Surgery,” American Journal of Sports Medicine 46 (2018): 890–899. 29364702 10.1177/0363546517749834 PMC7263374

[37] H. K. Kim , S. Shiraj , C. G. Anton , P. S. Horn , and B. J. Dardzinski , “Age and Sex Dependency of Cartilage T2 Relaxation Time Mapping in MRI of Children and Adolescents,” American Journal of Roentgenology 202 (2014): 626–632. 24555601 10.2214/AJR.13.11327

[38] J. B. Catterall , T. V. Stabler , C. R. Flannery , and V. B. Kraus , “Changes in Serum and Synovial Fluid Biomarkers After Acute Injury (NCT00332254),” Arthritis Research & Therapy 12 (2010): R229. 21194441 10.1186/ar3216 PMC3046542

[39] E. Berzolla , V. Sundaram , B. A. Lezak , et al., “Synovial Fluid Biomarkers in the Contralateral Knee Predict Patient‐Reported Outcomes After Injury at Long‐Term Follow‐Up,” American Journal of Sports Medicine 54 (2026): 278–284. 41626727 10.1177/03635465251401555

[40] E. Harris , “Study: Waist‐to‐Hip Ratio Might Predict Mortality Better Than BMI,” Journal of the American Medical Association 330 (2023): 1515–1516. 10.1001/jama.2023.19205 37792387

[41] D. Gallagher , S. B. Heymsfield , M. Heo , S. A. Jebb , P. R. Murgatroyd , and Y. Sakamoto , “Healthy Percentage Body Fat Ranges: An Approach for Developing Guidelines Based on Body Mass Index,” American Journal of Clinical Nutrition 72 (2000): 694–701. 10966886 10.1093/ajcn/72.3.694

