# Supplementary material for: Associations Between Patient Characteristics and Cartilage T1ρ Relaxation Times Vary Over Time Following Patellar Dislocation
Source: J Orthop Res. 2026 Jun 9;44(6):e70233. doi: 10.1002/jor.70233 (PMC13248002; doi:10.1002/jor.70233)
Supplement: Supplementary file 1 — Table S1: Average (± standard deviation) T1ρ relaxation times for all regions of cartilage for all groups. [file JOR-44-0-s001.docx]

| Table S-1: Average (± standard deviation) T1ρ relaxation times for all regions of cartilage for all groups. | | | | |
| --- | --- | --- | --- | --- |
|  | Baseline Injured | Baseline Contralateral | Follow-Up Injured | Follow-Up Contralateral |
| Medial Patella | 42.2±7.6 | 38.9±5.3 | 40.3±4.7 | 39.8±5.8 |
| Central Patella | 41.6±5.9 | 41.4±4.0 | 41.0±3.6 | 41.5±4.9 |
| Lateral Patella | 40.2±4.9 | 40.2±3.6 | 39.6±2.8 | 39.9±3.6 |
| Medial Trochlear Groove | 43.4±4.9 | 43.6±4.1 | 43.3±3.1 | 43.6±3.5 |
| Central Trochlear Groove | 44.1±3.8 | 44.2±3.7 | 43.9±3.2 | 44.3±3.2 |
| Lateral Trochlear Groove | 44.5±4.4 | 44.6±3.8 | 44.2±3.2 | 44.4±2.7 |
| Medial Femoral Condyle | 45.1±4.5 | 45.1±4.2 | 44.1±3.7 | 44.9±3.9 |
| Medial Tibia | 41.0±3.5 | 41.0±4.2 | 40.0±4.4 | 40.7±4.3 |
| Lateral Femoral Condyle | 44.4±3.8 | 43.8±3.8 | 43.6±3.0 | 43.7±2.8 |
| Lateral Tibia | 38.8±3.0 | 38.6±3.6 | 37.9±3.4 | 38.8±3.3 |
